# Vital personality scores and healthy aging: Life-course associations and familial transmission

**DOI:** 10.1016/j.socscimed.2021.114283

**Published:** 2021-09

**Authors:** Jasmin Wertz, Salomon Israel, Louise Arseneault, Daniel W. Belsky, Kyle J. Bourassa, HonaLee Harrington, Renate Houts, Richie Poulton, Leah S. Richmond-Rakerd, Espen Røysamb, Terrie E. Moffitt, Avshalom Caspi

**Affiliations:** a Department of Psychology and Neuroscience, Duke University, Durham, NC, USA; b Department of Psychology, The Hebrew University of Jerusalem, Jerusalem, Israel; c Institute of Psychiatry, Psychology & Neuroscience, King's College London, London, UK; d Mailman School of Public Health, Columbia University, New York, USA; e Dunedin Multidisciplinary Health and Development Research Unit, Department of Psychology, University of Otago, Dunedin, New Zealand; f Department of Psychology, University of Oslo, Oslo, Norway; g Norwegian Institute of Public Health, Norway; h Center for Genomic and Computational Biology, Duke University, Durham, NC, USA; i Department of Psychiatry and Behavioral Sciences, Duke University, Durham, NC, USA; j Department of Psychology, University of Michigan, Ann Arbor, MI, USA; k Duke University Medical Center, Center for the Study of Aging and Human Development, USA

**Keywords:** Personality, Mortality, Health, Life-course, Big 5

## Abstract

**Objectives:**

Personality traits are linked with healthy aging, but it is not clear how these associations come to manifest across the life-course and across generations. To study this question, we tested a series of hypotheses about (a) personality-trait prediction of markers of healthy aging across the life-course, (b) developmental origins, stability and change of links between personality and healthy aging across time, and (c) intergenerational transmission of links between personality and healthy aging. For our analyses we used a measure that aggregates the contributions of Big 5 personality traits to healthy aging: a “vital personality” score.

**Methods:**

Data came from two population-based longitudinal cohort studies, one based in New Zealand and the other in the UK, comprising over 6000 study members across two generations, and spanning an age range from birth to late life.

**Results:**

Our analyses revealed three main findings: first, individuals with higher vital personality scores engaged in fewer health-risk behaviors, aged slower, and lived longer. Second, individuals’ vital personality scores were preceded by differences in early-life temperament and were relatively stable across adulthood, but also increased from young adulthood to midlife. Third, individuals with higher vital personality scores had children with similarly vital partners, promoted healthier behaviors in their children, and had children who grew up to have more vital personality scores themselves, for genetic and environmental reasons.

**Conclusion:**

Our study shows how the health benefits associated with personality accrue throughout the life-course and across generations.

## Highlights

• A multi-trait index of ‘vital personality’ was derived.
• Individuals with higher vital personality scores aged more slowly.
• Individuals with higher vital personality scores had healthier lifestyles.
• Parents with higher vital personality scores promoted healthier habits in their children.
• Findings replicated in two cohorts.

## 1 Introduction

Life expectancy has been increasing around the world, fuelling research efforts to understand determinants of healthy aging. There is growing recognition of the role of psychological factors in healthy aging (Carter et al., 2019; Miething et al., 2020), including of people's personalities. A wealth of research now documents links between personality traits, aging, and mortality (Allen and Laborde, 2017; Graham et al., 2017; Israel et al., 2014; Jokela et al., 2013; Luchetti et al., 2014). In order to translate these findings into interventions to delay aging, more work is needed to understand how associations between personality traits and aging unfold across the life-course. Here we addressed this question.

### 1.1 Conceptual framework

Our work was guided by two overarching conceptual frameworks. The first is a life-course perspective on aging. It conceptualizes aging as a lifelong process, recognizing that age-related morbidity and mortality can be predicted from early in life (Moffitt et al., 2017). The second is a life-course perspective on associations between personality and health and aging. This perspective is exemplified by models such as the life-course chain-of-risk model of personality and aging, which proposes that personality gives rise to a multifaceted cascade of health outcomes (e.g. personality - > smoking - > cardiovascular disease - > premature death) (Chapman et al., 2014), and the life-course of personality model (Shanahan et al., 2014) which conceptualizes personality as a predictor of lifelong health and aging, with effects that accumulate over the life-course and are mediated by multiple pathways within the individual (e.g. health behaviors) and their social environment (e.g. relationship and family outcomes) (Hampson and Friedman, 2008; Mroczek et al., 2020; Shanahan et al., 2014).

### 1.2 Hypotheses

Based on these frameworks and previous research, we tested three sets of hypotheses. First, we tested whether personality traits predicted individual differences in aging already in the first half of life. Previous research shows that personality traits are associated with age-related disease and mortality (Graham et al., 2017; Jokela et al., 2013, 2014; Terracciano et al., 2014). However, life-course chain-of-risk models predict that personality is associated with diverging aging trajectories even before differences in age-related disease and mortality become apparent (Chapman et al., 2014). A difficulty with testing this hypothesis is that there are few available indicators of aging in younger, still-healthy populations. Here we used a new biomarker-composite that captures a person's biological pace of aging by measuring within-individual physiological deterioration in multiple organ systems (Belsky et al., 2015, 2020). Even in relatively young adults (ages 26–38), this measure shows a pattern of age-dependent decline in bodily functioning (Belsky et al., 2015), and predicts future physical and cognitive deterioration (Belsky et al., 2020). In addition to the pace of aging, we also tested associations with health habits, which are a key precursor of poor health outcomes in models of personality-health associations, such as the chain-of-risk model and health-behavior models of personality (Smith, 2006; Turiano et al., 2015). We focused on modifiable health habits shown to predict aging, including smoking, alcohol consumption, physical inactivity and stress-coping strategies (Hampson et al., 2007; Mroczek et al., 2009).

Second, we examined the developmental origins and stability of links between personality and aging across time. Precursors of individual differences in personality traits are apparent from early in life, as predicted by models of personality development (Caspi and Shiner, 2007); and evident in a wealth of research documenting associations between early-life temperament and adult personality (Caspi et al., 2005; McAdams and Olson, 2010). This suggests that the personality ‘ingredients’ for healthy aging can be identified early in life, which has implications for the targeting of aging interventions. Here we directly tested this hypothesis, as well as stability and change in aging-related personality variation over time once children grew into adulthood.

Third, we studied family origins of links between personality and healthy aging. Social-relations models and social-ecological models of health emphasize the importance of the social environment for one's health (McLeroy et al., 1988; Tay et al., 2013), including family members. We studied family member resemblance in personality and health among spouses, and among parents and children. Previous research reports resemblance in personality traits among spouses (Rammstedt et al., 2013), which may have implications for the health of each spouse (Ruiz et al., 2006). Previous work also shows that personality traits are transmitted across generations (Bouchard and McGue, 2003), suggesting that the processes underlying associations between personality and healthy aging in one generation are set into motion in earlier generations; consistent with the life-course of personality model (Shanahan et al., 2014). Furthermore, research shows that children grow up in families that differ in the extent to which they promote the development of healthy behaviours (Loprinzi and Trost, 2010; Rossow and Rise, 1994). We used personality data from mothers, fathers and children to test the familial aggregation and transmission of links between personality and healthy aging.

### 1.3 Multi-cohort approach

A challenge to tracing associations between personality and healthy aging across the life-course is that few studies have followed up individuals from birth to old age. We addressed this challenge by combining data from two cohorts of individuals of different ages, and, in each cohort, from individuals and their parents. Individuals were participants of two population-representative, longitudinal cohort studies, one in New Zealand, of participants followed from birth to midlife, and the other in the UK, of twins followed from birth to early adulthood. Both cohorts have had high retention (95 % and 93 %, respectively). In both studies, we measured variation in five dimensions of personality, which have been labeled the “Big Five” because they provide a replicable, parsimonious description of differences in individuals’ personalities across samples, raters, and cultures: Openness, Conscientiousness, Extraversion, Agreeableness and Neuroticism (Digman, 1990; McCrae and Costa, 2008). We used data from these different cohorts and generations to expand the range of the life-course that was covered by our samples and to conduct a conceptual replication of our findings (i.e. an attempt to replicate findings using different data or methods; Pridemore et al., 2018). Participants in the cohorts were born 20 years and 20,000 km apart and analyses used groups ranging from young children to older adults, making for a good replication check. Taken together, our samples total over 6000 individuals and cover an age range from birth to late life (Table 1).

**Table 1:** Description of samples included in the analyses.

	Dunedin participants	Dunedin parents	E-Risk participants	E-Risk parents	Total
Sample size (n)^a^	1037	1717	2232	1986	6972
Age range covered (years)	0–38	47–83	0–18	20–63	0–83
Analyses that use sample					
H1: Mortality risk		×			
H2: Pace of Aging	×				
H3: Health habits	×		×		
H4: Childhood temperament	×		×		
H5: Relative stability	×				
H6: Mean-level change	×				
H7: Partner resemblance		×		×	
H8: Intergenerational resemblance	×	×	×	×	
H9: Heritability			x		
H10: Health-parenting				×	

a For Dunedin and E-Risk participants, this is the total number of participants in each sample. The effective number of participants included in our analyses varies due to missing data and is reported for each analysis.

### 1.4 The “vital personality” score

To capture aging-related variation across Big 5 personality traits for all participants regardless of age, we constructed a multi-trait measure that quantifies the cumulative contribution of an individual's Big 5 personality traits to aging. To do so, we borrowed methods from algorithm-based scoring systems that have been used to summarize risk for clinical disease, e.g. the Framingham Risk Score (Wilson et al., 1998), and genetic risk, i.e. polygenic risk scores (Dudbridge, 2013). These algorithms construct individual scores to predict risk of an outcome (e.g., cardiovascular disease) by weighting multiple factors (e.g., health-risk factors or gene-variants) by the strength of association with the outcome, as estimated in independent training samples (e.g., a health survey or genome-wide association study). We adopted this approach for three reasons. First, it is well-suited to summarize associations between multiple predictors (e.g. Big 5 traits) and an outcome. For example, the construction of the multi-index Framingham Risk score was partly motivated by the observation that cardiovascular disease was predicted by multiple personal characteristics (D'Agostino et al., 2013). For associations between the Big 5 traits and aging, a similar picture emerges, with links between several Big 5 traits and markers of health and aging such as health behaviors (Hakulinen et al., 2015a,b; Hakulinen et al., 2015a,b; Strickhouser et al., 2017; Wilson and Dishman, 2015), inflammation (Armon et al., 2013), chronic disease (Jokela et al., 2014; Terracciano et al., 2014), and mortality (Graham et al., 2017; Jokela et al., 2013). Second, the scoring approach can increase generalizability and reproducibility, because it uses a pre-specified set of predictors and an external scoring key (i.e. weights estimated in an independent training sample). It can also increase usability if the external scoring key is easily available; and acceptability of the resulting score, because the score calculation is not tied to a specific theory but is based on an external standard (such as a meta-analysis). Third, this approach calibrates a score to a specific outcome of interest, by weighing each contributing factor by its association with that outcome. Constructing a score based on weights estimated for a specific outcome steers the score in the direction of an interpretation of the resulting measure.

We used the published regression weights from a meta-analysis of associations between Big 5 traits and mortality (Jokela et al., 2013) to develop a poly-trait measure for mortality, or as we call it: a “vital personality” score. This score aggregates the contributions of the Big 5 personality traits to mortality. The approach of computing personality profiles using meta-analytic results has been used in research on obesity (Vainik et al., 2019) and attainment (Mõttus et al., 2017). Here we applied this method to studying associations between personality and aging. We used the results of a meta-analysis as our external scoring key, because a meta-analysis estimates associations across many different samples, individuals, and methods of assessing personality; thus, robustness and reproducibility are built into the vital personality score. We used a meta-analysis of mortality as our scoring algorithm because the age of one's death is a relatively unbiased indicator of (un)healthy aging, reflecting a person's illness burden accumulated from health-risk factors across the life-course.

In the meta-analysis, each Big 5 trait apart from Openness was individually associated with mortality, with effect sizes ranging from HR = 0.87 (for Conscientiousness) to HR = 1.08 (for Neuroticism) (Jokela et al., 2013; Web Table 3). For our vital personality score we included all Big 5 traits, and opted against modeling nonlinearities and intercorrelations, to keep the score calculation as simple as possible; avoid introducing score idiosyncrasies that could be based on possibly unique interaction or correlation patterns in our data; and keep with practices of previous score construction approaches, e.g. for polygenic scores (Dudbridge, 2013). Before using the vital personality score in our analyses, we validated it by testing whether individuals with higher vital personality scores had a lower mortality risk (as expected based on the meta-analysis on which the score is based) (H1). We then used the vital personality score to test the hypotheses outlined above, about (a) associations between personality and biological aging and personality and health habits, predicting that a higher vital personality score would be associated with less accelerated biological aging (H2) and better health habits (H3); (b) the development of vital-personality variation across the life-course, predicting that childhood temperament would be associated with adult vital personality scores (H4) and testing the extent of rank-order (H5) and mean-level stability of vital personality (H6); and (c) familial origins and intergenerational transmission of the vital personality score, predicting that spouses (H7) and parents and children (H8) would resemble each other in vital personality scores; exploring the relative contributions of genetic and environmental influences on vital personality scores (H9); and predicting that parents’ vital personality scores would be associated with their greater promotion of offspring health habits (H10). We report additional analyses using each of the Big 5 traits individually (rather than the aggregate vital personality score) in the supplementary materials.

**Table 2:** Childhood temperament predicts adult vital personality scores.

	Dunedin cohort		E-Risk cohort	
Predictors	β	(95 % CI)	β	95 % CI
Lack of control	**-.21**	**(-.27, -.15)**	**-.**09	**(-.14, -.04)**
Sluggishness	**-.12**	**(-.18, -.06)**	**-.06**	**(-.11, -.02)**
Approach	.06	(.00, .13)	**.09**	**(.04, .14)**

*Note.* The table shows standardized regression coefficients (β) estimated in separate models predicting vital personality scores (measured at age 26 in Dunedin and age 18 in E-Risk) from childhood temperament (measured at ages 3 and 5 years in Dunedin and at age 5 years in E-Risk). Analyses are adjusted for sex. Bolded estimates are statistically significant (p < .01). Dunedin n = 943; E-Risk n = 2046.

**Table 3:** Mothers with higher vital personality scores promoted healthier habits in their children.

	Model 1^a^	Model 2^b^
Health-parenting outcome	β (95 % CI)	β (95 % CI)
Hours children watch TV per day	**- .2**2 **(-.28, -.16)**	**- .21 (-.27, -.15)**
Hours children play videogames per day	**- .19 (-.26, -.13)**	**- .1**9 **(-.2**6**, -.1**3**)**
Times children eat fruits and vegetables per week	**.**19 **(.13, .25)**	**.18 (.12, .25)**
Times children eat takeaway foods per week	**- .14(-.2**0**, -.**08**)**	**- .14(-.21, -.08)**
How often twins brush teeth	**.20 (.14, .27)**	**.18 (.12, .2**5**)**
Times children eat crisps or sweets per week	- .01 (−.07, .06)	- .01 (−.07, .06)

*Note.* For all outcomes apart from brushing teeth, the response options were in units of x per day or week. For brushing teeth, the scale ranged from 0 (Never) to 5 (Three times per day). Bold estimates are statistically significant (p < .05). Estimates are adjusted for sex. Analyses were restricted at the family-level to one randomly-selected twin. The n for each analysis varied from 977 to 988 depending on item. CI=Confidence interval. β = Standardized estimate.

a Model 1: Predictors include maternal vital personality score only.

b Model 2: Predictors additionally include study members' own vital personality score, measured at age 18.

## 2 Methods

### 2.1 Samples

This study used data from two population-based longitudinal cohort studies; the Dunedin Longitudinal Study in New Zealand, and the Environmental Risk (E-Risk) Longitudinal Twin Study in the UK. In both studies, we collected data from participants and their parents (Table 1).

Dunedin participants (*N* = 1037; 91 % of eligible births; 52 % male) were all individuals born between April 1972 and March 1973 in Dunedin (NZ), who were eligible based on residence and participated in the first assessment at age 3 (Poulton et al., 2015). The cohort represented the full range of SES in the general population of New Zealand's South Island. On adult health, it matches the NZ National Health & Nutrition Survey (Poulton et al., 2015). The cohort is primarily white (93 %). Assessments were carried out at birth and ages 3, 5, 7, 9, 11, 13, 15, 18, 21, 26, 32, and 38 years, when 95 % (*n* = 961) of the 1007 participants still alive took part. There were no statistically significant differences between the full cohort and those seen at age 38 on childhood SES, childhood IQ and childhood behavioral problems (all *p*s > .05). At assessments, each study member is brought to the research unit for a day of interviews and examinations. The Otago Ethics Committee approved each phase of the study. Data on Dunedin participants' parents were collected during home visits carried out from 2003 to 2006 when participants were 30–33 years old (Milne et al., 2008). 94 % (n = 945) of living Dunedin participants had at least one parent participate; 72 % (n = 723) had two. The total number of individual parents for whom information was available was n = 1717 (n = 1625 biological-parents).

The E-Risk sample was constructed in 1999–2000, when N = 1116 families (93 % of those eligible) with same-sex 5-year-old twins (56 % monozygotic, 44 % dizygotic; sex was evenly distributed within zygosity; 49 % male) participated in home-visit assessments. The cohort represents the full range of socioeconomic conditions in Great Britain (Odgers et al., 2012a, 2012b). The cohort is primarily white (90 %). Home visits were conducted with children and mothers at ages 7, 10, 12, and with children only at 18 years, when 93 % (*n* = 2066) of participants took part. There were no statistically significant differences between the full cohort and those seen at age 18 on childhood SES, childhood IQ and childhood behavioral problems (all *p*s > .05). The Joint South London and Maudsley and the Institute of Psychiatry Research Ethics Committee approved each phase of the study. Data on E-Risk participants' parents were collected during the childhood home visits. Interviews were conducted with mothers, and mothers reported on children's fathers. 99 % of participants had a mother participate in the study; 80 % (*n* = 1796) had information about both parents. The total number of individual parents for whom information was available was *n* = 1986. All mothers and most fathers (89 %; *n* = 801) were biological-parents.

### 2.2 Personality measures

We measured the personalities of Dunedin and E-Risk participants through reports by co-informants (mostly best friends, partners, or other family members) as previously described (Israel et al., 2014; Richmond-Rakerd et al., 2019). Reports were made on a brief, 25-item version of the Big 5 Inventory (Benet-Martínez and John, 1998) measuring the personality traits of openness to experience (“Original, comes up with new ideas”), conscientiousness (“Works until a thing is done”), extraversion (“Outgoing, likes people”), agreeableness (“Kind and considerate”), and neuroticism (“Gets nervous easily”). We measured the personalities of participants’ parents through reports by trained research workers who conducted structured interviews with the parents, or, for E-Risk fathers, through reports by E-Risk mothers, using the same 25-item Big 5 inventory as for participants (Benet-Martínez and John, 1998). Internal-consistency reliabilities and intercorrelations of the Big 5 scales in each sample are reported in Supplementary Table S1; the median reliability across all samples was α = 0.76).

### 2.3 Health and aging outcomes

*Mortality.* We measured mortality of Dunedin participants' biological parents through participants' reports about their parents’ death, up to 2019. Among the 1505 biological parents for whom personality and mortality data were available, there were 182 parental deaths (12 %).

*Pace of Aging*. We measured the pace of biological aging of Dunedin participants as changes in 18 biomarkers of cohort members' cardiovascular, metabolic, endocrine, pulmonary, hepatic, renal, immune, and periodontal systems across ages 26, 32, and 38 years (Belsky et al., 2015). A detailed description is available in the Supplement (Supplementary Text 1). The measure quantifies participants’ rate of aging in year-equivalent units of physiological decline per chronological year. The average study member experienced 1 year of physiological decline per year, a Pace of Aging of 1. The fastest-aging participants experienced more than twice this rate of change, while the slowest-aging participants experienced almost no change (Belsky et al., 2015; Supplementary Text 1).

*Health habits*. We measured health habits (smoking, drinking, physical activity, stress-coping strategies) in Dunedin and E-Risk participants through structured interviews. In both cohorts, smoking was measured as number of cigarettes smoked per day; drinking was measured as average number of alcoholic drinks per week; leisure physical activity was assessed as time spent on physically demanding activities per week and stress-coping strategies were assessed by asking participants how often they used each of a list of strategies to cope with stress (e.g., “Talk with other people”; “Give up”; Table S3). We created measures of ‘active’, ‘passive’ and ‘distressed’ coping based on factor analysis (Tables S3). Means and standard deviations are reported in Supplementary Tables S3 and S6.

### 2.4 Life-course antecedents and correlates

*Childhood temperament*. We measured Dunedin and E-Risk participants' temperament during childhood (age 3 years in Dunedin; age 5 years in E-Risk), through reports by trained research workers as previously described (Caspi et al., 1995). Research workers rated each child-participant on 22 adjectives items on a scale from 0 ‘not at all’ to 2 ‘definitely’. Items loaded on three replicable factors: “lack of control” indexing emotional lability and short attention span (example items: ‘lability’; ‘easily frustrated’, ‘impulsivity’); “sluggishness” indexing shyness and fear (example items: ‘flat affect’; ‘passivity’; ‘self-criticism’); and “approach” indexing self-confidence, and self-reliance (example items: ‘friendliness’; ‘quick adjustment’; ‘self-confidence’) (Caspi et al., 1995). Internal-consistency reliabilities ranged from 0.65 to 0.90 in the Dunedin Study (Caspi et al., 1995) and 0.68 to 0.90 in E-Risk.

*Health parenting.* We measured E-Risk mothers' promotion of a healthy lifestyle for their children at the age-10 home visit. Mothers responded to items about children's health-related behaviors that are known to predict better health outcomes, such as how many hours they watched television per day (Robinson et al., 2017), how many servings of fruit and vegetables they ate per week (Hartley et al., 2013) and how often they brushed their teeth (Collett et al., 2016) (Table S4). Means and standard deviations are reported in Supplementary Table S4.

### 2.5 Construction of the vital personality score

For each study participant and each parent, a vital personality score was calculated as O × o + C × c + E × e + A × a + N × n where O, C, E, A, and N are the natural log of the hazard ratios for mortality risk associated with a 1 SD difference in each of the Big 5 (Openness to Experience [HR 0.94], Conscientiousness [HR 0.87], Extraversion [HR 0.91], Agreeableness [HR 0.94], and Neuroticism [HR 1.08]) as estimated in a previous, independent meta-analysis in 76,150 individuals (Jokela et al., 2013; Web Table 3), and o, c, e, a, and n are Big 5 personality factor *z*-scores for the individual study member. Vital personality scores were standardized (*M* = 0 and SD = 1) and reverse scored so that a higher score would be expected to predict better health and aging outcomes (Figure S1).

### 2.6 Statistical analyses

To test associations between Dunedin parents’ vital personality scores and mortality, we used Cox proportional hazards models adjusted for sex and age at baseline and standard errors adjusted for the clustering of parents within participants. To test associations between vital personality scores and other variables (i.e. Pace of Aging; Smoking; Drinking; Exercise; Coping; Childhood temperament) we used linear regression models. When analyzing E-Risk twin data, standard errors were adjusted for clustering of twins within families. To test associations between the vital personality score and health-risk behaviors within twins, we used sibling fixed-effects regression models to account for family-wide factors shared between siblings growing up in the same family. To test rank-order stability of the vital personality score over time, we used linear regression models. To test mean-level change over time, we used longitudinal growth models. All models were adjusted for sex. To test the heritability of the vital personality score, we used biometric twin models that decompose variance in an outcome into additive genetic influences (A), shared-environmental influences (C) that make members of a family similar, and non-shared environmental influences (E) that make members of a family different, including measurement error. There were few missing data points in our sample due to high participation and retention rates in both studies (Dunedin retention at age 38: 95 %; E-Risk retention at age 18: 93 %). We used listwise deletion to deal with missing data. In analyses where missing data led to the exclusion of 10 or more participants on the predictor variables, we conducted sensitivity analyses using full-information maximum likelihood estimation to deal with missing data; this did not change our findings (Supplementary Text 2). We do not report *R*^2^ for each individual model, but report standardized effect estimates, which can be squared to obtain the amount of variance that the vital personality score explains in each outcome.

We used Stata version 14.1. (StataCorp, 2015), and OpenMx for R (version 2.19.6; Boker et al., 2011). Analyses were independently checked for reproducibility by a data-analyst using SAS version 9.4 (SAS Institute Inc, 2013) and MPlus version 8.3. (Muthén & Muthén, 1998–2017).

## 3 Results

### 3.1 Vital personality scores were associated with lifespan and healthspan

**Fig. 1:**
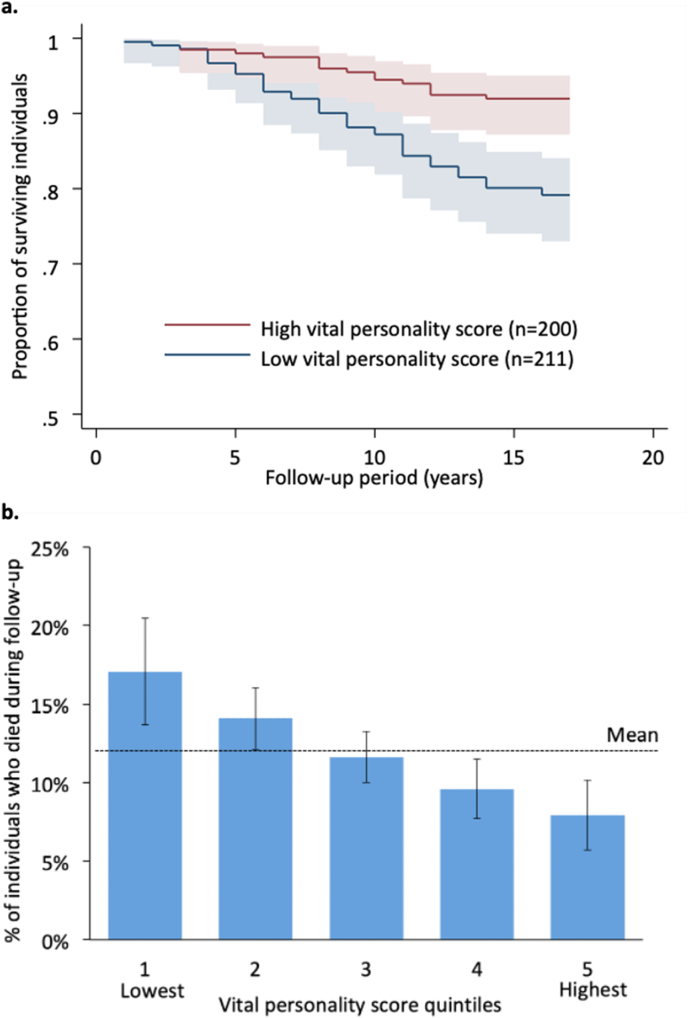
Participants with higher vital personality scores lived longer. *Note.* The figures show associations between vital personality score and mortality in Dunedin participants' biological parents. **Panel a.** shows Kaplan Meier survival functions of the proportion of surviving individuals by follow-up year, separately for parents with high vital personality scores (score ≥ 1 SD above the mean; n = 200) versus parents with low vital personality scores (score ≤ 1 SD below the mean; n = 211). At the time of personality assessment, Dunedin biological parents' ages ranged from 47 to 83 years (M = 58, SD = 5). We focused on deaths that occurred between the years when parents' personalities were assessed (2003–2006) and 2019. The plots show that individuals with higher vital personality scores at baseline stayed alive for longer. Estimates are adjusted for parents' sex and age at baseline. n = 1497. **Panel b.** shows the per cent of individuals who died during follow-up by vital personality score quintile. Estimates are adjusted for parents' sex and age at baseline. Shaded areas and error bars indicate 95 % confidence intervals. Supplementary data shows associations between the vital personality score and mortality when removing one Big 5 trait at a time (Table S5a) and between each Big 5 trait and mortality separately (Table S5b).

Hypothesis 1 **Vital personality scores are associated with lower mortality risk***.* Dunedin parents' vital personality scores were associated with their mortality risk after adjusting for baseline age and for sex. A 1 SD increase in vital personality score was was associated with a 25 % lower risk of death during follow-up (HR = .75, 95 % CI [.67, .84], p < .01) Fig. 1a). The difference in mortality among individuals in the bottom versus top quintiles of the vital personality score amounted to 9 percentage points (17.1 % versus 7.9 %; Fig. 1b). The vital personality score was not dependent on any single personality dimension for predicting mortality (Table S5a). Notably, vital personality scores were associated with mortality after removing Conscientiousness (HR = .82, 95 % CI [.73, .93], *p* < .01), the Big 5 dimension carrying the largest weight in the score.

Hypothesis 2 **Vital personality scores are associated with slower aging***.* Dunedin participants' vital personality scores at age 26 years were associated with slower biological aging during their 30s (β = -.16, 95 % CI [-.22, −.09], *p* < .01, *n* = 921). The measure is scaled so that the average study member experiences 1 year of physiological decline per chronological year; a Pace of Aging of 1. Study members with the highest vital personality scores (i.e. highest quintile) aged 0.94 biological years per chronological year between ages 26–38 years, compared to 1.10 biological years per chronological year for study members with the lowest vital personality scores (i.e. lowest quintile). This amounted to approximately 2 fewer years of biological aging between ages 26 to 38 for participants in the top vs bottom quintiles on vital personality score.

**Fig. 2:**
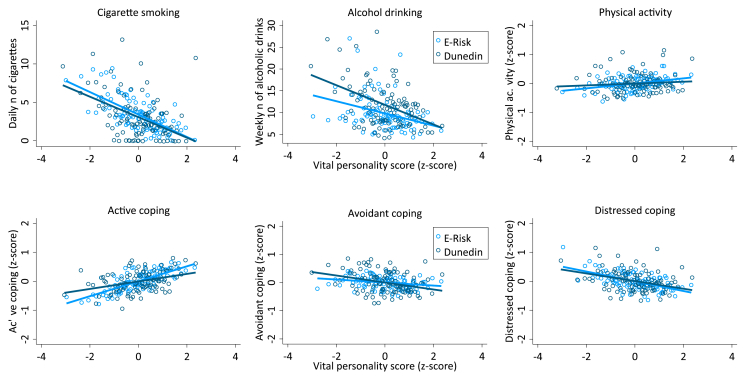
Individuals with higher vital personality scores tended to have halthier lifestyles, in both the Dunedin and E-Risk cohorts. *Note.* The figures show sex-adjusted associations between vital personality scores and health habits in the Dunedin (dark-blue) and E-Risk (light-blue) cohorts. N's were as follows: Smoking (Dunedin n = 920; E = Risk n = 2038); Drinking (Dunedin n = 914; E-Risk n = 2036); Exercise (Dunedin n = 918; E-Risk n = 2035); Coping (Dunedin n = 913; E-Risk n = 2037). Each plotted point represents mean x and y coordinates for a bin of about 10 Dunedin and 20 E-Risk participants. Regression lines were estimated from unbinned data. All measures apart from physical activity were assessed in the same way in both cohorts, so associations can be compared across cohorts. Table S6 reports descriptives, whole-sample estimates in both samples and within-twin-pair estimates based on twins in the E-Risk cohort. (For interpretation of the references to colour in this figure legend, the reader is referred to the Web version of this article.)

Hypothesis 3 **Vital personality scores are associated with leading healthier lives*****.*** Individuals with higher vital personality scores tended to have healthier lifestyles. Dunedin participants with higher vital personality scores at age 26 years tended to smoke fewer cigarettes β = −.19, 95 % CI [-.25, −.12], *p* < .01), drink less alcohol (β = −.10, 95 % CI [-.17, −.04], *p* < .01) be more physically active (β = .09, 95 % CI [.02, .15], *p* < .01), and to use more active (β = .12, 95 % CI [.06, .19], *p* < .01), less avoidant (β = −.12, 95 % CI [-.18, −.05], *p* < .01) and less distressed coping strategies at age 38 (β = −.16, 95 % CI [-.23, −.10], *p* < .01) (Fig. 2). Effect sizes were small. Findings replicated among 18-year old E-Risk participants with mostly similar effect sizes (Fig. 2, Table S6) and even within E-Risk families: compared to their co-twins, E-Risk participants with higher vital personality scores tended to smoke fewer cigarettes, to be more active, and to use more active stress-coping strategies, but did not differ in other stress-coping strategies or alcohol consumption (Table S6).

*Additional analysis: How does the vital personality score compare to using each Big 5 trait separately as a predictor?* For our main health outcomes (mortality, pace of aging, health habits) we compared estimates when using the vital personality score versus each Big 5 trait separately (Tables S5b and S7). We note three findings. First, the vital personality score showed consistent associations with most outcomes, whereas individual-trait associations were less consistent. Second, associations tended to be larger when the vital personality score was used as a predictor, compared to using one Big 5 trait at a time. Third, whereas order and magnitude of individual-trait associations differed across cohorts, those between the vital personality score and most outcomes were remarkably similar.

### 3.2 Vital personality scores across the life-course

Hypothesis 4 **Vital personality scores are preceded by differences in childhood temperament***.* In both cohorts, participants' behavioral styles in childhood forecast their vital personality scores in adulthood. Those who showed greater control, less sluggishness and (in E-Risk) greater approach as children, grew up to have higher vital personality scores (Table 2). Effect sizes were small.

Hypothesis 5 **Individual differences in vital personality score are stable*****.*** Vital personality scores showed considerable rank-order stability from age 26 to age 38 years, implying that Dunedin participants with relatively higher vital personality scores at one age tended to have higher vital personality scores at later ages (correlations ranged from *r* = .54 to *r* = .61 across ages, *n* = 874).

Hypothesis 6 **Vital personality scores increase with age***.* There was a small mean-level increase in participants' vital personality scores over the adult years, as indicated by the linear slope estimate of a longitudinal growth model fitted to repeated measures of Dunedin participants' personality traits at ages 26, 32, and 38 years (β = .008, 95 % CI [.003, .013], *p* < .05, *n* = 847). This amounted to approximately a one tenth of a standard deviation increase in the vital personality score from age 26 to age 38. Further testing indicated that this increase was entirely explained by a rise in participants' conscientiousness (Figure S2).

### 3.3 Vital personality scores in the family

**Fig. 3:**
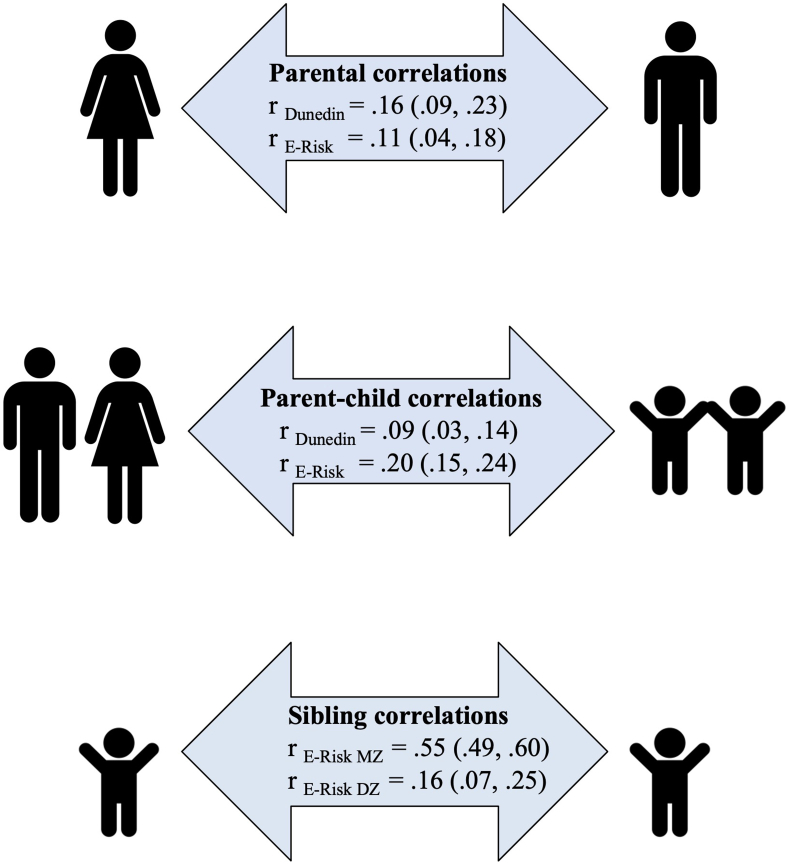
Vital personality scores are correlated within families. *Note.* The figure shows correlations in vital personality scores between Dunedin and E-Risk participants' parents (top arrow, Dunedin dyads n = 740; E-Risk n = 897); participants' parents and participants (i.e. parent and child; middle arrow, Dunedin families n = 902; E-Risk n = 1011); and between identical (MZ) and nonidentical (DZ) twin participants in the E-Risk cohort (bottom arrow, MZ n = 574, DZ n = 440). 95 % Confidence intervals are reported in parentheses. Estimates from analyses in only-biological parents were very similar (Table S10).

Hypothesis 7 **Partners resemble each other in their vital personality scores*****.*** The personalities of romantic partners resembled each other in vitality, as indicated by small positive correlations in vital personality scores between the mothers and fathers of Dunedin and E-Risk participants (Fig. 3).

Hypothesis 8 **Vital personality scores are correlated across generations*****.*** Parents with higher vital personality scores tended to have children who grew up to also have higher vital personality scores, as indicated by small positive correlations in vital personality scores between parents and (adult) children in both cohorts (Fig. 3). These results did not differ for biological vs non-biological parents (Table S8).

Hypothesis 9 **Vital personality scores are heritable*****.*** Individual differences in vital personality scores were partly attributable to genetic influences, as indicated by E-Risk identical (MZ) twin correlations more than twice as high as non-identical (DZ) correlations (Fig. 3). Formal twin modeling indicated that 53 % of variance in age-18 vital personality was accounted for by genetic influences (A = .53, 95 % CI [.47, .58]). Environmental influences shared between twins did not account for any variance (C = .00 [95 % CI .00, .05]). The remaining variance was accounted for by environmental influences not shared between twins, including measurement error (E = .47, 95 % CI [.42, .53]).

Hypothesis 10 **Parents with higher vital personality scores promote children’s healthier living***.* The children of E-Risk mothers with higher vital personality scores tended to spend fewer hours watching television and playing videogames; eat fruits and vegetables more often and takeaway food less often; and brush their teeth more often; effect sizes were similar across these outcomes (Table 3). There were no differences in how often children ate crisps and sweets. Re-analyzing all associations controlling for offspring's own vital personality scores did not change these results (Table 3).

## 4 Discussion

Our in-depth investigation of associations between vital personality scores and healthy aging across the life-course and across generations revealed three main findings. First, individuals with higher vital personality scores tended to lead healthier lives from a young age. As children, they tended to eat healthier, spent less time in front of screens, and brushed their teeth more often. As young adults, they tended to smoke and drink alcohol less; to be more physically active; and to use healthier stress-coping strategies. By mid-life, they were more likely to have formed relationships with similarly vital partners, and they tended to age at a slower pace than their cohort peers with lower vital personality scores. Second, individuals’ vital personality scores showed both stability and change across development. The developmental origins of a higher vital personality score were evident as early as age 3, in measurements of childhood temperament. Across the adult years, individuals tended to retain the placement of their vital personality score within the population. Third, individuals with higher vital personality scores made decisions that were associated not only with their own health, but also that of future generations. Individuals with higher vital personality scores tended to select partners with higher vital personality scores, and their children tended to go on to have higher vital personality scores themselves. As parents, those with higher vital personality scores promoted healthier habits of their children, building a foundation for health in the next generation.

One of the aims of our study was to test whether a vital personality score – a multi-trait score ‘calibrated’ to predict mortality -- would be associated with health indicators and behaviors that lie on the path toward a longer lifespan, such as pace of aging and health habits. We could not directly test this hypothesized mediation because the participants in our two main studies, Dunedin and E-Risk, are still young and few have died. However, in this young, healthy population we find, as hypothesized, that the vital personality score is already associated with possible precursors to longer lifespan such as accelerated biological aging.

Our findings raise the question: why is the vital personality score associated with slower aging and lower mortality risk? Our findings suggest several explanations. First, individuals with higher vital personality scores led healthier lives. They smoked and drank less, exercised more, and used more active stress-coping strategies. Such behaviors are predictors of age-related disease and mortality and mediators of personality-health associations (Jokela et al., 2013; Mroczek et al., 2009; Turiano et al., 2015). Second, associations between the vital personality score and biological aging may partly reflect genetic influences. For example, previous research suggests that genetic influences that contribute to individual differences in personality traits might also contribute to longevity (Bae et al., 2013). Third, individuals with higher vital personality scores were surrounded by parents, partners, and children with higher vital personality scores. Although we found no evidence for familial social transmission in the form of shared-environmental influences, individuals with higher vital personality scores might still affect each others' health (Bourassa, Knowles, Sbarra, & O'Connor, 2016; Ruiz et al., 2006).

Our study shows familial resemblance in the vital personality score, as evidenced by (a) correlations in vital personality scores of parents; (b) correlations in vital personality scores of parents and their offspring; and (c) correlations in vital personality scores of siblings growing up in the same family. Resemblance in parents' vital personality scores could be due to a tendency to select partners who have traits similar to one's own (i.e., assortative mating; Botwin et al., 1997); non-random remaining in relationships (Caspi and Herbener, 1990; Rammstedt et al., 2013); or mutual influences on health behaviors and personality over time (Ask et al., 2013). Resemblance in parent-offspring and siblings' vital personality scores could be due to genes, or due to family members influencing each other in ways that increase personality similarity. Our findings showed genetic influences on vital personality scores, and no evidence for shared-environmental influences, consistent with previous research (Bouchard and McGue, 2003). We did observe large estimates of non-shared environmental influences, indicating that environments might be more important in explaining why family members differ rather than resemble each other in their personalities (Plomin, 2011).

The vital personality score that we used in our study was constructed using meta-analytic weights of five Big 5 traits (Jokela et al., 2013). There are other ways to construct a multi-trait personality score, for example by adding up the Big 5 personality traits instead of weighing each trait; by weighing each trait by its unique association with mortality (i.e. adjusting for the intercorrelations between Big 5 traits), by constructing a higher-order factor of personality based on intercorrelations of the Big 5 (Digman, 1997; Musek, 2007) or by identifying people with different profiles using procedures such as latent profile analysis. We chose the approach used in this study because it complements other approaches, with some unique advantages. Constructing a score based on previously estimated weights is an empirically-keyed solution, and therefore minimizes the risk of overfitting a factor to the structure of a particular cohort and allows better comparison across samples. Constructing a score using results of a meta-analysis means that estimates are based on analyses of many different samples, individuals and methods of assessing personality, and may therefore improve generalizability and reproducibility. Constructing a score based on weights estimated for a specific phenotype steers the score in the direction of an interpretation, i.e. in our case it can be interpreted as a mortality-relevant personality trait (Mõttus et al., 2017).

The vital personality score could be used in follow-on research. There is long-standing interest in capturing combinations of Big 5 traits (Asendorpf, 2002; Robins et al., 1998) associated with health. An example is the literature on personality types linked with health, such as the “Type A” personality and cardiovascular disease (Smith et al., 2012). However, there is a lack of consensus about which profiles or types to use; a patchy replication history of specific profiles; and a pattern of inconsistent associations between personality profiles and health (Costa et al., 2002; Ioannidis, 2012; Myrtek, 1995). The vital personality score addresses some of these concerns and represents a straightforward and parsimonious way to represent personality contributions to healthy aging. As such, it could be used by aging researchers across disciplines who are looking to incorporate personality measures into their studies. For these researchers, the aggregate nature of the vital personality score might be an advantage. Furthermore, because the vital personality score is not tied to the assumptions of a specific theory (other than the Big 5 model), and based on an external scoring key, it can be flexibly used to test predictions of different theoretical frameworks linking psychological variables to health and mortality.

The vital personality score could also be used in healthcare, to support the move towards a more personalized medicine. Greater integration of social, psychological, and behavioral data with conventional clinical data could help clinicians better understand the needs of their patients, facilitate coordinated action, and increase patient-centredness (Israel and Moffitt, 2014; Matthews et al., 2016). For this promise to be realized, there is a need for standardized, easy-to-use, parsimonious measures of the psychosocial determinants of health. The vital personality score responds to this demand.

A question is whether the vital personality score provides any advantage over using each Big 5 trait separately. We addressed this question in our study, showing that associations between personality and aging tended to be larger when the vital personality was used, compared to using any other trait by itself. The findings also show that associations between the vital personality score and most outcomes were remarkably consistent across two cohorts of participants who grew up in different countries and in whom outcomes had been assessed at different ages. In contrast, the order and magnitude of individual-trait associations showed much greater variation across cohorts. These findings suggest that the aggregate nature of the vital personality delivers greater replicability of personality-health associations across study samples.

### 4.1 Limitations

Our findings should be interpreted in light of limitations. First, not all outcomes (e.g. mortality) were measured in the same sample. Second, we used short measures to assess Big 5 traits, and informants varied across samples (co-informants for participants; research workers for parents). The convergent results across informants support the validity and replicability of our findings and suggest that findings carry over to contexts (e.g., the clinic) in which extensive Big 5 measurement is difficult. Third, the vital personality score does not include information about different facets of the Big 5 traits, which may differ in their prediction of mortality. Fourth, the vital personality score is an aggregate score and does not provide inferences about which specific traits mediate health-associations. Fifth, the meta-analysis that provided the scoring weights for the vital personality score was based on primarily white study populations from Western countries; more research in non-white and non-Western populations is needed to test generalizability. Sixth, reliabilities for our heterogeneous coping scales were low, which may have attenuated associations with coping. Seventh, effect sizes were uniformly small. However, many effect sizes were on par with those of more established predictors of healthy aging, including SES; smoking; self-reported health and family medical history (Heistaro et al., 2001; Israel et al., 2014; Roberts et al., 2007).

## 5 Conclusions

Our findings reveal that personality processes associated with healthy aging, as captured by the vital personality score, are evident from before birth and early in life onwards. If these associations turn out to be causal, it underscores the need to start early with interventions to extend healthy lives (Kuh, 2007) and suggests that such efforts have pay-offs across generations (Heckman and Karapakula, 2019). The findings also point to the many possible mediators of personality-aging associations across the life-course (Friedman et al., 2014; Jokela et al., 2013; Mroczek et al., 2009; Turiano et al., 2015). To identify these mediators, it is critical to study individuals while they are still free from age-related disease and follow them as they develop and change (Kuh, 2007; Moffitt et al., 2017; Shanahan et al., 2014). Taken together, these findings emphasize the importance of a life-course approach to studying aging, to elucidate pathways toward healthy long lives.
