# Supplementary material for: Vital personality scores and healthy aging: Life-course associations and familial transmission
Source: Soc Sci Med. 2021 Sep;285:114283. doi: 10.1016/j.socscimed.2021.114283 (PMC8482063; doi:10.1016/j.socscimed.2021.114283)
Supplement: Multimedia component 1 [file mmc1.docx]

SUPPLEMENTARY MATERIALS

“Vital personality scores and healthy aging: Life-course associations and familial transmission”

**Supplementary Text 1**

Measurement of pace of biological aging

We measured Pace of Aging as previously described (Belsky et al., 2015), from repeated assessments of a panel of 18 biomarkers: Glycated hemoglobin, Forced expiratory volume in one second (FEV1), Blood pressure (mean arterial pressure), Total cholesterol, C-reactive protein, Creatinine clearance, Urea nitrogen, Cardiorespiratory fitness (VO2Max), Waist-hip ratio, Forced vital capacity ratio (FEV1/FVC), Body mass index (BMI), Leukocyte telomere length (LTL), Lipoprotein(a), Triglycerides, Periodontal disease, White blood cell count, High density lipoprotein (HDL), and Apolipoprotein B100/A1 ratio. Biomarkers were assayed at the age-26, -32, and -38 assessments. (Albumin, Alkaline phosphatase, and Cytomegalovirus IgG could not be included in the Pace of Aging because they were measured only at the age-38 assessment.)

We calculated each Study member’s Pace of Aging in three steps. In the first step, we transformed the biomarker values to a standardized scale. For each biomarker, we standardized values according to the age-26 distribution, setting the mean to zero and the corresponding standard deviation to one. Standardization was conducted separately for men and women. Scores were reversed for VO2Max, FEV1/FVC, FEV1, LTL, creatinine clearance, and HDL cholesterol, which are known to decline with age. Thus, standardized biomarker values greater than zero indicated levels that were “older” and values less than zero indicated levels “younger” as compared to the average 26-year-old. Over the 12 years of follow-up, the biomarker panel indicated a progressive deterioration of physiological integrity with advancing chronological age; i.e. values tended to increase from the age-26 assessment to the age-38 assessment.

In the second step, we calculated each Study member’s personal slope for each of the 18 biomarkers—the average year-on-year change observed over the 12-year period. Slopes were estimated using a mixed effects growth model that regressed the biomarker level on age.

In the third step, we combined information from the slopes of the 18 biomarkers to calculate each Study member’s personal “pace of aging.” Because we did not have a priori basis for weighting differential contributions of the biomarkers to an overall pace of aging measure, we combined information using a unit-weighting scheme. (All biomarkers were standardized to have mean=0, SD=1 based on their age-26 distributions, so slopes were denominated in comparable units). We calculated each study member’s Pace of Aging as the sum of age-dependent annual changes in biomarker Z-scores. Pace of Aging was normally distributed in the cohort (M=0.70 age-26 SD units, SD=0.29).

Because the Dunedin birth cohort represents its population, its mean and distribution represent population norms. We used these norms to scale the Pace of Aging to reflect physiological change relative to the passage of time. We set the cohort mean Pace of Aging as a reference value equivalent to the physiological change expected during a single chronological year. Using this reference value, we rescaled Pace of Aging in terms of years of physiological change per chronological year. On this scale, cohort members ranged in their Pace of Aging from near zero years of physiological change per chronological year to nearly three years of physiological change per chronological year.

Measurement of health-parenting

As part of the age-10 home visit, mothers responded to items querying them about health-related behaviors they required of their children. Items were selected based on querying health psychologists and conducting focus groups with mothers. We do not report reliability estimates for these items because they are single items. There is high face validity for these items, exact wording as follows:

- “How many hours of television do the twins watch on an average day?” (answer options are 0, 1, 2, 3, 4, 5, 6, 7, 8, 9, 10, 11, 12)
- “How many hours do they spend playing video games on an average day?” (answer options are 0, 1, 2, 3, 4, 5, 6, 7, 8, 9, 10, 11, 12)
- “How often do the twins eat fresh fruit and vegetables in a week?” (answer options are 0, 1, 2, 3, 4, 5, 6, 7, 8, 9, 10, 11, 12, 13, 14)
- “How often do the twins eat takeaway food in a week?” (answer options are 0, 1, 2, 3, 4, 5, 6, 7, 8, 9, 10, 11, 12, 13, 14)
- “How many times in a week do the twins eat crisps or sweets?” (answer options are 0, 1, 2, 3, 4, 5, 6, 7, 8, 9, 10, 11, 12, 13, 14)
- “How often do they brush their teeth?” (answer options are 0 ‘never’; 1 ‘once a week; 2 ‘a few times a week’; 3 ‘once a day’; 4 ‘twice a day’; 5 ‘three times a day’; 6 ‘four times or more each day’)

**Supplementary Text 2**

Sensitivity analyses

In response to reviewer comments, we conducted two types of sensitivity analyses. First, we re-ran analyses where there were more than n=10 missing data points on the predictor variables. We used full-information maximum likelihood estimation as implemented in MPlus version 8.3. (Muthén & Muthén, 1998-2017) to deal with missing data in these analyses. This approach did not substantively change our findings. For associations between the vital personality score and pace of aging, estimates changed from (β =-.16 [95%CI -.22; -.09], p<.01, n=921) to (β =-.16 [95%CI -.22; -.10], p<.01, n=954). For associations between vital personality scores across time, the correlation between age-26 and age-32 vital personality scores changed from β =.58 [95%CI .54; .62], p<.01, n=874 to β =.58 [95%CI .54; .63], p<.01, n=928; the correlation between age-32 and age-38 vital personality scores changed from β =.61 [95%CI .57; .64], p<.01, n=874 to β =.61 [95%CI .57; .65], p<.01, n=933 and the correlation between age-38 and age-26 vital personality scores changed from β =.54 [95%CI .49; .58], p<.01, n=874 to β =.53 [95%CI .48; .57], p<.01, n=933. For growth curve models estimating change in vital personality scores across time, the estimate changed from β=.008 [95%CI .003, .013], p<.05, n=847 to β=.007 [95%CI .002, .011], p<.05, n=933.

Second, we accounted for the low reliability in Dunedin fathers’ Big 5 scales (internal-consistency reliabilities were Openness=.87; Conscientiousness=.58; Extraversion=.60; Agreeableness=.79; Neuroticism=.81). We did this using a structural equation modeling approach, defining single-indicator latent variables for each Big 5 trait and fixing the residual variance to a value that reflects the reliability of the each measure (Brown, 2006). We then constructed the vital personality score using these variables. This approach did not substantively change the findings: estimates of the vital personality’s prediction of mortality in the Dunedin fathers changed from HR= .72, 95% CI [.62, .84], p<.01 to HR= .75, 95% CI [.65, .88], p<.01.

**Supplementary Table S1**. Internal-consistency reliabilities (Cronbach’s α) for Big 5 reports in Dunedin and E-Risk samples.

|  | Openness | Conscientiousness | Extraversion | Agreeableness | Neuroticism |
| --- | --- | --- | --- | --- | --- |
| **Dunedin participants^+^** |  |  |  |  |  |
| Age 26 (co-informant reports) | .85 | .82 | .79 | .76 | .84 |
| Age 32 (co-informant reports) | .81 | .76 | .70 | .72 | .79 |
| Age 38 (co-informant reports) | .79 | .74 | .70 | .71 | .75 |
| **Dunedin participant parents^v^** |  |  |  |  |  |
| Mothers (interviewer reports) | .86 | .67 | .65 | .69 | .86 |
| Fathers (interviewer reports) | .87 | .58 | .60 | .79 | .81 |
| **E-Risk participants^x^** |  |  |  |  |  |
| Age 18 (co-informant reports) | .80 | .78 | .68 | .66 | .70 |
| **E-Risk participant parents*** |  |  |  |  |  |
| Mothers (interviewer reports) | .91 | .86 | .78 | .78 | .83 |
| Fathers (mothers’ reports) | .75 | .67 | .66 | .71 | .76 |

*Note:* Personality measures were standardized to M=0, and SD=1 in our analyses. Because both our cohorts accurately represent the full variation in the populations from which they were drawn, z scores provide highly informative population norms.

**^+^** Dunedin participants’ personalities were measured through reports by co-informants on three occasions (ages 26, 32, and 38 years) as previously described (Israel et al., 2014). At each age, Dunedin study members nominated three people “who knew them well” (mostly best friends, partners, or family members). Co-informants were provided with questionnaires asking them to describe the study member using a brief, 25-item version of the Big 5 Inventory (Benet-Martínez & John, 1998) measuring the personality traits of openness to experience (“Original, comes up with new ideas”), conscientiousness (“Works until a thing is done”), extraversion (“Outgoing, likes people”), agreeableness (“Kind and considerate”), and neuroticism (“Gets nervous easily”). Complete Big 5 data were obtained for n=946 (97%) of participating study members at age 26; n=935 (96%) of participating study members at age 32; and n=933 (97%) of participating study members at age 38.

**^v^** Personalities of Dunedin participants’ parents were measured through reports by trained research workers when the parents were interviewed as part of the Dunedin Family Health History Study (Milne et al., 2008) in 2003-2006, when parents were, on average 58 years old (SD=5, range: 41-83). After the home visit, research workers rated the personalities of Dunedin study members’ parents using the same 25-item Big 5 inventory used for Dunedin study members (see description above). Complete Big 5 data were obtained for 99% (n=1,709) of the parents who participated in the Family History Study.

**^x^** E-Risk participants’ personalities were measured through reports by co-informants at age 18 as previously described (Richmond-Rakerd et al., 2019). Participants nominated two people “who knew them well” (mostly parents and co-twins). Co-informants were provided with questionnaires asking them to describe the study member using the same 25-item Big 5 inventory used for Dunedin study members (Benet-Martínez & John, 1998). Complete Big 5 data were obtained for 2,050 (99.2%) of participating study members at age 18.

***** Personalities of E-Risk participants’ mothers were measured through reports by trained research workers when study members were age 7 years old and the mothers were, on average 34 years old (SD=6, range=20-49). After the home visit, research workers rated the personalities of mothers using the same 25-item Big 5 inventory as used in the Dunedin study and for the E-Risk participants. Complete Big 5 data were obtained about n=1,087 mothers (100%) of those participating in the age-7 assessment. Personalities of E-Risk participants’ fathers were measured through reports by mothers, using the same instrument. We asked mothers to rate their children’s fathers, because many E-Risk households were single-parent families and because fathers are notoriously difficult to recruit into research (Mitchell et al., 2007). Complete Big 5 data were obtained for n=898 fathers.

**Supplementary Table S2**. Big 5 trait intercorrelations as estimated in the Dunedin and E-Risk cohorts, and, for comparison purposes, in two previous meta-analyses.

|  | Dunedin study members (age 26) | Dunedin mothers | Dunedin fathers | E-Risk study members (age 18) | E-Risk mothers | E-Risk fathers | Jokela et al.  (2013)  meta-analysis**^^^** | | van der Linden (2010)  meta-analysis**^+^** | |
| --- | --- | --- | --- | --- | --- | --- | --- | --- | --- | --- |
| Correlation | r | r | r | r | r | r | | r | | r |
| O and C | **.22** | **.23** | **.33** | **.28** | **.52** | **.39** | | **.22** | | **.14** |
| O and E | **.36** | **.47** | **.40** | **.21** | **.40** | **.38** | | **.35** | | **.31** |
| O and A | **.19** | **.13** | **.12** | **.26** | **.40** | **.25** | | **.20** | | **.14** |
| O and N | **- .18** | **- .28** | **- .12** | **- .09** | **- .25** | **- .17** | | **- .14** | | **- .12** |
| C and E | **.12** | .04 | .03 | .04 | **.19** | **.21** | | **.24** | | **.21** |
| C and A | **.35** | **.22** | **.21** | **.42** | **.48** | **.38** | | **.36** | | **.31** |
| C and N | **.22** | **- .32** | **- .10** | **- .10** | **- .30** | **- .24** | | **- .22** | | **- .32** |
| E and A | **.30** | **.19** | **.20** | **.24** | **.34** | **.29** | | **.29** | | **.18** |
| E and N | **- .16** | **- .26** | **- .26** | **- .23** | **- .36** | **- .15** | | **- .19** | | **- .26** |
| A and N | **- .38** | **- .35** | **- .48** | **- .31** | **- .35** | **- .41** | | **- .21** | | **- .26** |

*Note:* r=Pearson’s correlation coefficient. Bold estimates are statistically significant (p<.05). O=Openness to experience; C=Conscientiousness; E=Extraversion; A=Agreeableness; N=Neuroticism. Study members’ mothers and fathers’ include both biological and non-biological parents.

**^^^**These estimates come from Jokela et al., 2013, WebTable 2. They are pooled estimates across 7 population-based studies.

**+**These estimates come from van der Linden, te Nijenhuis, & Bakker, 2010, Table 2. They are meta-analytic Big 5 intercorrelations estimated from 212 studies.

**Supplementary Table S3**. Descriptive information about coping strategies included in the stress-coping measures in the Dunedin and E-Risk cohorts.

|  | Dunedin cohort | E-Risk cohort |
| --- | --- | --- |
|  | M (SD) | M (SD) |
| **Active coping strategies** |  |  |
| Talk with other people | 1.47 (.63) | 1.12 (.81) |
| Work more/work harder | 1.24 (.76) | .89 (.83) |
| Exercise | .69 (.79) | .78 (.85) |
| Take steps right away to solve the problem. | 1.58 (.58) | 1.01 (.78) |
| **Avoidant coping strategies** |  |  |
| Ignore problems/ put them out of mind | .62 (.70) | .60 (.74) |
| Focus on other things in your life | 1.04 (.70) | .93 (.79) |
| **Distressed coping strategies** |  |  |
| Obsess about problems | .70 (.73) | .51 (.76) |
| Withdraw / spend more time alone | .57 (.73) | .74 (.80) |
| Talk with a therapist/counsellor | .15 (.47) | .10 (.39) |
| Sleep more | .27 (.58) | .46 (.72) |
| Give up | .14 (.40) | .22 (.52) |

*Note:* M=Mean, SD=Standard Deviation. The stress-coping questionnaires included four additional strategies that were not included in these measures. “Drink alcohol” and “Smoke” were not included because they were already assessed separately. “Eat more” and “Pray/go to church/talk with a religious leader” were not included because they did not load clearly on any factor.

**Supplementary Table S4**. Descriptives of E-Risk study members’ mothers health-parenting.

|  | Descriptives |
| --- | --- |
| Health-parenting outcome**^+^** | M (SD) |
| Hours children watch TV per day | 2.66 (1.24) |
| Hours children play videogames per day | 1.18 (1.05) |
| Times children eat fruits and vegetables per week | 8.77 (4.19) |
| Times children eat takeaway foods per week | 0.74 (0.69) |
| How often twins brush teeth | 3.66 (.63) |
| Times children eat crisps or sweets per week | 6.16 (3.00) |

*Note*: M=Mean, SD=Standard Deviation

**+**We measured health parenting (i.e., the extent to which parents promote a healthier lifestyle for their children) in the E-Risk cohort, through mothers’ reports at age 10 years. As part of the age-10 home visit, mothers responded to items querying them about health-related behaviors they required of their children, as listed in the table. For all outcomes apart from brushing teeth, the response options were in units of x per day or week. For brushing teeth, the scale ranged from 0 (Never) to 5 (Three times per day).

**Supplementary Table S5**. Estimates of associations between the vital personality score and mortality, leaving out one Big 5 trait at a time (**Panel A**), and between each Big 5 trait with mortality separately (**Panel B**).

| **PANEL A** | HR (95%CI) |  | **PANEL B** | HR (95%CI) |
| --- | --- | --- | --- | --- |
| Including all five traits in the vital personality score (as done in our main analyses) | **.75 (.67, .84)** |  | Including all five traits in the vital personality score (as done in our main analyses) | **.75 (.67, .84)** |
| Leaving out openness | **.76 (.69, .85)** |  | Openness by itself | **.78 (.67, .91)** |
| Leaving out conscientiousness | **.82 (.73, .93)** |  | Conscientiousness by itself | **.76 (.68, .85)** |
| Leaving out extraversion | **.75 (.67, .83)** |  | Extraversion by itself | .90 (.78, 1.03) |
| Leaving out agreeableness | **.74 (.66, .84)** |  | Agreeableness by itself | **.85 (.76 .95)** |
| Leaving out neuroticism | **.73 (.65, .81)** |  | Neuroticism by itself | 1.07 (.93, 1.23) |

*Note:* HR= Hazard ratio; CI=Confidence interval. Bold estimates are statistically significant (p<.05).

**Supplementary Table S6**. Estimates of associations between the vital personality score and health habits (health-risk behaviors, i.e. smoking, drinking, exercise; and stress-coping strategies, i.e. active, avoidant and distressed coping) in Dunedin and E-Risk participants and in E-Risk twins.

|  | Dunedin participants | E-Risk participants | Within E-Risk  twin pairs |
| --- | --- | --- | --- |
|  | Estimate (95%CI) | Estimate (95%CI) | Estimate (95%CI) |
| **Health habits^+^** |  |  |  |
| Cigarette smoking | **-.19 (-.25, -.12)** | **-.20 (-.24, -.15)** | **-.10 (-.15, -.04**) |
| Alcohol drinking | **-.10 (-.17, -.04)** | **-.08 (-.12, -.04)** | -.06 (-.13, .02) |
| Exercising | **.09 ( .02, .15)** | **.10 ( .05, .14)** | **.13 ( .06, .19)** |
| **Stress-coping strategies^*^** |  |  |  |
| Active coping | **.12 ( .06, .19)** | **.26 ( .22, .30**) | **.14 ( .07, .21)** |
| Avoidant coping | **-.12 (-.18, -.05)** | -.05 (-.10, .00) | -.02 (-.10, .05) |
| Distressed coping | **-.16 (-.23, -.10)** | **-.18 (-.22, -.13)** | -.05 (-.12, .02) |

*Note:* CI=Confidence interval. Bold estimates are statistically significant (p<.05). All estimates are based on standardized data and adjusted for sex (apart from the within-twin-pair models, because these use data from same-sex twins).

**^+^** Health habits were measured through structured interviews with 38-year old Dunedin participants and 18-year old E-Risk participants. Smoking was measured as the number of cigarettes participants reported smoking per day (Dunedin: M = 3.11, SD = 6.85; E-Risk: M = 3.48, SD = 7.49). Alcohol consumption was measured as the average number of alcoholic drinks participants reported consuming per week (Dunedin: M = 12.17, SD = 16.79; E-Risk: M = 9.91, SD = 16.48). In Dunedin, leisure physical activity was measured using reports of the number of minutes study members reported engaging in different types of physically demanding activities during an average week. Time spent on each activity was converted to metabolic equivalent (MET) units (Ainsworth et al., 2000). We summed weekday and weekend METs from moderate (e.g., walking the dog) or more strenuous (e.g., cycling) leisure activities to calculate physical activity levels (M=17.9, SD=22.6). In E-Risk, leisure physical activity was measured using the Stanford Brief Activity Survey (Taylor-Piliae et al., 2010). Study members selected one of five patterns of physical activity that best described the way they spent their leisure time, ranging from inactive (score 1; watching television, reading, playing on the computer or phone) to very hard intensity (score 5; engaging in regular program of physical fitness such as jogging or running on a daily basis) (M=2.38, SD=1.26).

**^*^** Coping strategies were measured through structured interviews with 38-year old Dunedin participants and 18-year old E-Risk participants. In Dunedin, participants were asked how they cope with stress in two different contexts: with finances and at work (e.g., “When you feel stressed about your finances, which of the following things do you do to cope?”). Participants were shown a list of coping strategies (e.g., “Talk with other people”; “Take steps right away to solve the problem”; “Give up”; **Table S3**) and indicated their response as 0 ‘not true’, 1 ‘somewhat true’ or 2 ‘very true’. Responses were averaged across contexts. In E-Risk study members, the same instrument was used as in the Dunedin study, without referring to a specific context (i.e., “Lots of different things can make you feel stressed out, for example; finances, relationships, college exams or work.”). We used exploratory factor analysis in each cohort to create three summary measures of items that showed consistent loadings across the two cohorts: ‘active coping’; ‘avoidant coping’; and ‘distressed coping’; **Table S3).** The correlations were: active and avoidant coping, Dunedin r=.09, E-Risk r=.02; active and distressed coping Dunedin r=.10, E-Risk r=-.07; avoidant and distressed coping Dunedin r=.13, E-Risk r=.11). Internal-consistency reliabilities (Cronbach’s alpha α) for the coping checklist scales were: active coping (Dunedin α=.39; E-Risk α=.48); passive coping (Dunedin α=.68; E-Risk α=.54); distressed coping (Dunedin: α=.56; E-Risk α=.49).

**Supplementary Table S7**. Comparison of associations between the vital personality versus each Big 5 trait separately with the pace of aging and health-lifestyle outcomes in the Dunedin and E-Risk cohorts.

|  | **Dunedin cohort** | | | | | | |
| --- | --- | --- | --- | --- | --- | --- | --- |
|  | Pace of Aging | Smoking | Drinking | Exercise | Active coping | Avoidant coping | Distressed coping |
|  | Estimate (95%CI) | Estimate (95%CI) | Estimate (95%CI) | Estimate (95%CI) | Estimate (95%CI) | Estimate (95%CI) | Estimate (95%CI) |
| Vital personality | **-.16 (-.22,.-09)** | **-.19 (-.25,-.12)** | **-.10 (-.17,-.04)** | **.09 (.02, .15)** | **.12 ( .06, .19)** | **-.12 (-.18, -.05)** | **-.16 (-.23, -.10)** |
| Openness | **-.10 (-.16,-.04)** | -.06 (-.12, .00) | -.07 (-.13, .00) | .05 (-.01, .12) | **.11 ( .04, .17)** | -.06 (-.13, .00) | .01 (-.06, .07) |
| Conscientiousness | **-.15 (-.21,-.08)** | **-.14 (-.20,-.08)** | -.06 (-.12, .01) | .02 (-.05, .08) | .05 (-.02, .11) | **-.12 (-.19,-.06)** | **-.10 ( .16,-.03)** |
| Extraversion | -.04 (-.10, .03) | -.04 (-.11, .03) | -.04 (-.10, .03) | **.11 ( .04, .17)** | **.15 ( .08, .21)** | -.05 (-.12, .01) | -.06 (-.13, .00) |
| Agreeableness | **-.08 (-.15,-.02)** | **-.17 (-.23,-.11)** | **-.12 (-.19,-.06)** | .03(-.03, .09) | **.08 ( .02, .14)** | -.03 (-.10, .03) | **-.13 (-.20,-.07)** |
| Neuroticism | **.11 ( .05, .18)** | **.18( .12, .24)** | .07 (.01, .14) | **-.08(-.14,.-.01)** | -.03 (-.09, .04) | .06 ( .00, .13) | **.23 ( .17, .30)** |
|  |  | | | | | | |
|  | **E-Risk cohort** | | | | | | |
|  |  | Smoking | Drinking | Exercise | Active coping | Avoidant coping | Distressed coping |
|  |  | Estimate (95%CI) | Estimate (95%CI) | Estimate (95%CI) | Estimate (95%CI) | Estimate (95%CI) | Estimate  (95%CI) |
| Vital personality |  | **-.20 (-.24,-.15)** | **-.08 (-.12,-.04)** | **.10 ( .05,.14)** | **.26 (.22,.30)** | -.05 (-.10, .00) | **-.18 (-.22, -.13)** |
| Openness |  | **-.11 (-.15,-.07)** | -.06 (-.11,-.02) | -.04 (-.08, .01) | **.11 ( .07, .16)** | -.02 (-.06, .03) | **.08 ( .03, .13)** |
| Conscientiousness |  | **-.21 (-.26,-.16)** | **-.14 (-.18,-.11)** | **.06 ( .01, .10)** | **.22 ( .18, .27)** | **-.11 (-.15,-.06)** | **-.13 (-.17,-.08)** |
| Extraversion |  | **.07 ( .02, .11)** | **.13 ( .08, .17)** | **.11 ( .07, .15)** | **.13 ( .09, .18)** | .04 ( .00, .09) | -.05 (-.10,-.01) |
| Agreeableness |  | **-.20 (-.24,-.15)** | **-.11 (-.16,-.06)** | .05 ( .00, .10) | **.18 ( .13, .22)** | -.05 (-.09, .00) | **-.17 (-.22,-.13)** |
| Neuroticism |  | **.13 ( .09, .18)** | .03 (-.01, .07) | **-.09 (-.13,-.04)** | **-.10 (-.14,-.05)** | -.02 (-.06, .03) | **.23 ( .19, .29)** |

*Note*: CI=Confidence interval. Bold estimates are statistically significant (p<.05). All estimates are based on standardized data and adjusted for sex. Bold estimates are statistically significant (p<.05). The shaded rows indicate the estimates between the vital personality score and outcomes.

**Supplementary Table S8**. Comparison of associations between parents’ vital personality scores and outcomes in biological and social versus biological parents only.

|  | In biological and  social parents | In biological parents only |
| --- | --- | --- |
|  | r (95%CI) | r (95%CI) |
| **Dunedin cohort** |  |  |
| Partners resemble each other in their vital personality | **.16 (.09, .23)** | **.16 (.09, .23)** |
| Vital personalities have vital children | **.09 (.03, .14)** | **.10 (.05, .16)** |
| **E-Risk cohort** |  |  |
| Partners resemble each other in their vital personality | **.11 (.04, .18)** | **.11 (.04, .18)** |
| Vital personalities have vital children | **.20 (.15, .24)** | **.21 (.16, .26)** |

*Note:* r=Pearson’s correlation coefficient. Bold estimates are statistically significant (p<.05).

**Supplementary Figure S1.** Vital personality score distribution in Dunedin (a) and E-Risk participants (b).

**a.**

**b.**

*Note:* The Figure depicts the distribution of the Vital personality score in the Dunedin (Panel a) and E-Risk (Panel b) cohorts. The y-axis of the figure shows the frequency of each score in the cohorts. The x-axis of the figure shows vital personality z-scores (one unit corresponds to one standard deviation).

**Supplementary Figure 2.** Mean-level change in Big 5 traits and vital personality score across ages 26, 32 and 38.

*Note:* All variables were standardized to the same scale using a z-score transformation anchored to the age-26 mean and standard deviation so that at age-26, each personality factor had M=0, SD=1. The mean values at age-32 and age-38 thus reflect average change in vital personality scores from the age-26 assessment. Formal testing using longitudinal growth models indicated that mean-level change was statistically significant at p<.05 for Conscientiousness, Extraversion and the Vital Personality score (p<.05), but not for any of the other traits. Mean-level change of the Vital Personality score was no longer statistically significant when Conscientiousness was excluded from the score.

**References**

Ainsworth, B. E., Haskell, W. L., Whitt, M. C., Irwin, M. L., Swartz, A. M., Strath, S. J., … Leon, A. S. (2000). Compendium of physical activities: An update of activity codes and MET intensities. *Medicine and Science in Sports and Exercise*, *32*. https://doi.org/10.1097/00005768-200009001-00009

Belsky, D. W., Caspi, A., Houts, R., Cohen, H. J., Corcoran, D. L., Danese, A., … Moffitt, T. E. (2015). Quantification of biological aging in young adults. *Proceedings of the National Academy of Sciences of the United States of America*, *112*, E4104–E4110. https://doi.org/10.1073/pnas.1506264112

Benet-Martínez, V., & John, O. P. (1998). Los Cinco Grandes across cultures and ethnic groups: Multitrait multimethod analyses of the Big Five in Spanish and English. *Journal of Personality and Social Psychology*, *75*, 729–750. Retrieved from http://www.ncbi.nlm.nih.gov/pubmed/9781409

Brown, T. A. (2006). *Confirmatory factor analysis for applied research*. New York, NY: The Guilford Press.

Israel, S., Moffitt, T. E., Belsky, D. W., Hancox, R. J., Poulton, R., Roberts, B., … Caspi, A. (2014). Translating personality psychology to help personalize preventive medicine for young adult patients. *Journal of Personality and Social Psychology*, *106*, 484–498. https://doi.org/10.1037/a0035687

Jokela, M., Batty, G. D., Nyberg, S. T., Virtanen, M., Nabi, H., Singh-Manoux, A., & Kivimäki, M. (2013). Personality and all-cause mortality: Individual-participant meta-analysis of 3,947 deaths in 76,150 adults. *American Journal of Epidemiology*, *178*, 667–675. https://doi.org/10.1093/aje/kwt170

Milne, B. J., Moffitt, T. E., Crump, R., Poulton, R., Rutter, M., Sears, M. R., … Caspi, A. (2008). How should we construct psychiatric family history scores? A comparison of alternative approaches from the Dunedin Family Health History Study. *Psychological Medicine*, *38*, 1793–1802. https://doi.org/10.1017/S0033291708003115

Mitchell, S. J., See, H. M., Tarkow, A. K. H., Cabrera, N., McFadden, K. E., & Shannon, J. D. (2007). Conducting studies with fathers: Challenges and opportunities. *Applied Developmental Science*, *11*, 239–244. https://doi.org/10.1080/10888690701762159

Muthén, L. K., & Muthén, B. O. (n.d.). *Mplus User’s Guide. Eighth Edition.* Los Angeles, CA: Muthén & Muthén.

Richmond-Rakerd, L. S., Caspi, A., Arseneault, L., Baldwin, J. R., Danese, A., Houts, R. M., … Moffitt, T. E. (2019). Adolescents who self-harm and commit violent crime: Testing early-life predictors of dual harm in a longitudinal cohort study. *American Journal of Psychiatry*, appi.ajp.2018.1. https://doi.org/10.1176/appi.ajp.2018.18060740

Taylor-Piliae, R. E., Fair, J. M., Haskell, W. L., Varady, A. N., Iribarren, C., Hlatky, M. a, … Fortmann, S. P. (2010). Validation of the Stanford Brief Activity Survey: examining psychological factors and physical activity levels in older adults. *Journal of Physical Activity & Health*, *7*, 87–94. https://doi.org/10.14440/jbm.2015.54.A

van der Linden, D., te Nijenhuis, J., & Bakker, A. B. (2010). The general factor of personality: A meta-analysis of big five intercorrelations and a criterion-related validity study. *Journal of Research in Personality*, *44*, 315–327. https://doi.org/10.1016/j.jrp.2010.03.003
